# Genome-scale reconstruction of the sigma factor network in *Escherichia coli*: topology and functional states

**DOI:** 10.1186/1741-7007-12-4

**Published:** 2014-01-24

**Authors:** Byung-Kwan Cho, Donghyuk Kim, Eric M Knight, Karsten Zengler, Bernhard O Palsson

**Affiliations:** 1 Department of Bioengineering, University of California, San Diego, La Jolla, CA 92093, USA; 2 Current address: Department of Biological Sciences, Korea Advanced Institute of Science and Technology, Daejeon 305-751, Republic of Korea; 3 Novo Nordisk Foundation Center for Biosustainability, Technical University of Denmark, Lyngby, Denmark

**Keywords:** *Escherichia coli*, Sigma factor, Network reconstruction, Comparative analysis, *Klebsiella pneumoniae*, Omics data, Systems biology

## Abstract

**Background:**

At the beginning of the transcription process, the RNA polymerase (RNAP) core enzyme requires a σ-factor to recognize the genomic location at which the process initiates. Although the crucial role of σ-factors has long been appreciated and characterized for many individual promoters, we do not yet have a genome-scale assessment of their function.

**Results:**

Using multiple genome-scale measurements, we elucidated the network of σ-factor and promoter interactions in *Escherichia coli*. The reconstructed network includes 4,724 σ-factor-specific promoters corresponding to transcription units (TUs), representing an increase of more than 300% over what has been previously reported. The reconstructed network was used to investigate competition between alternative σ-factors (the σ^70^ and σ^38^ regulons), confirming the competition model of σ substitution and negative regulation by alternative σ-factors. Comparison with σ-factor binding in *Klebsiella pneumoniae* showed that transcriptional regulation of conserved genes in closely related species is unexpectedly divergent.

**Conclusions:**

The reconstructed network reveals the regulatory complexity of the promoter architecture in prokaryotic genomes, and opens a path to the direct determination of the systems biology of their transcriptional regulatory networks.

## Background

**Figure 1:**
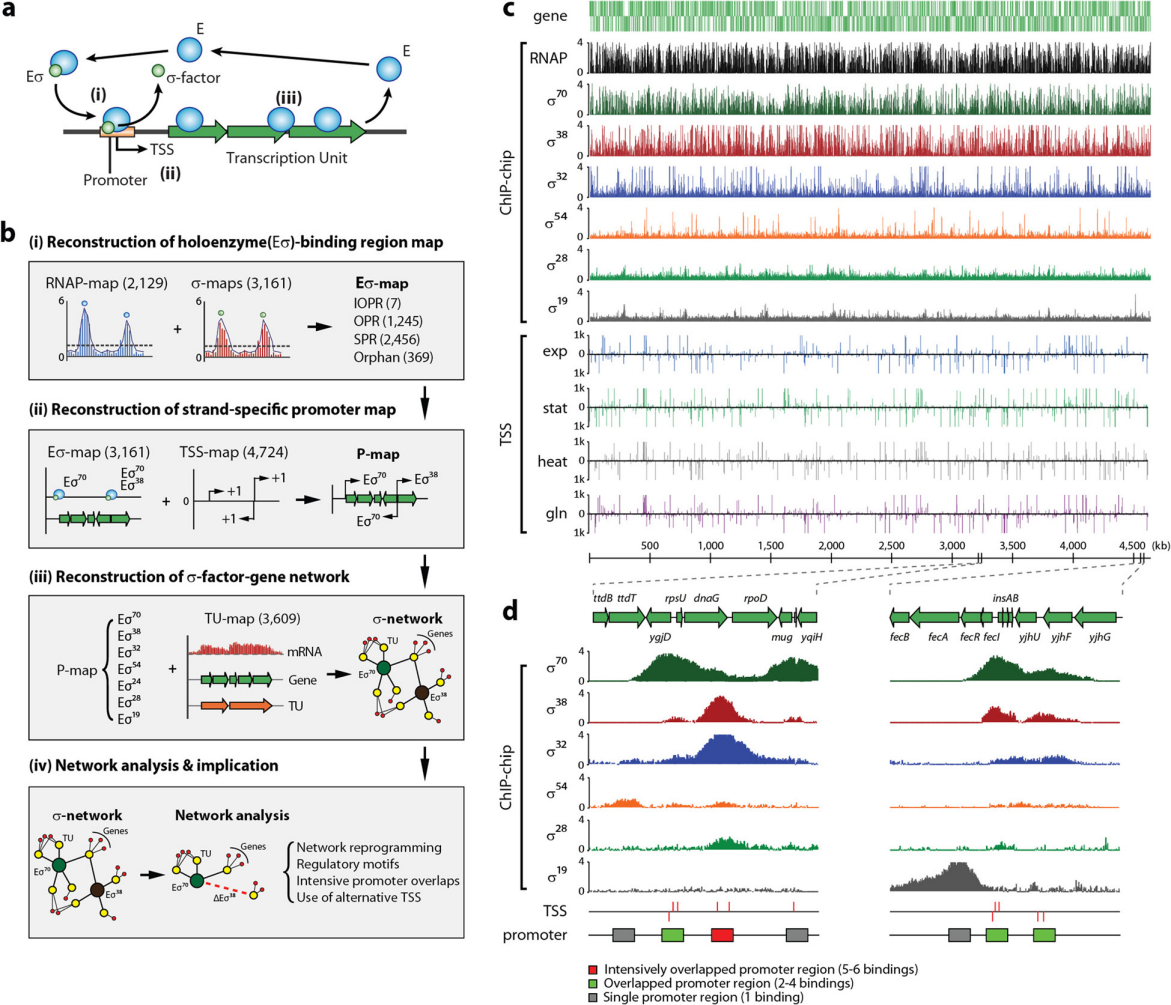
**Molecular basis of transcription and a reconstruction of σ-factor-transcription unit gene (σ-TUG) network from multi-omic experimental datasets. (a)** Diagram shows bacterial transcription process by an RNA polymerase (RNAP) core enzyme and an associated σ-factor. **(b)** Four-step process of multi-omic data integration to reconstruct the σ-TUG network. First, we identified RNAP-binding regions (RNAP map) and σ-factor binding regions (σ map) from RpoB and σ-factor chromatin immunoprecipitation and microarray (ChIP-chip) data (the missing σ^24^ binding information was taken from a public database [6]), resulting in the genome-wide holoenzyme binding map (Eσ map). The Eσ map was then combined with experimental transcription start site (TSS) information (TSS map), resulting in he strand-specific promoter map (P-map), which was integrated with previously reported TU information [7], resulting in the σ-network. With this σ-network, we then performed further analysis, such as network reprogramming, motif analysis, promoter overlapping, and alternative TSS usage. Subfigure I: IOPR, intensively overlapped promoter region; OPR, overlapped promoter region; SPR, single promoter region; Orphan, orphan promoter region. Subfigure III and IV: green and brown circles represent σ^70^ and σ^38^, yellow circles represent TUs, and red dots represent genes. Edges show regulatory interactions between elements. **(c)** Datasets used for σ-TUG network reconstruction: ChIP-chip dataset with RNAP and six σ-factors, and the TSS dataset. The TSS dataset for exponential phase was taken from a previous study [9].TSS subpanel: exp, exponential phase; stat, stationary phase; heat, heat shock; gln, alternative nitrogen source with glutamine. **(d)** Magnified examples of *rpoD* (left panel, genomic region ranging from 3,196 to 3,214 kbp), *fecI* and *fecRAB* (right panel, genomic region ranging from 4,494 to 4,517 kbp).

The RNA polymerase (RNAP) core enzyme (E) for bacterial transcription is a catalytic multi-subunit complex (α_2_ββ′ω), capable of transcribing portions of the DNA template into RNA transcripts. At the beginning of the transcribing process, the RNAP core enzyme requires a σ-factor to recognize the genomic location at which the process initiates [1-3] (Figure 1a). Then σ-factor, a single dissociable subunit, binds to E, forming a holoenzyme (Eσ^x^, x for each σ-factor) and orchestrates initiation of promoter-specific transcription [1]. To date, one housekeeping σ-factor σ^70^ (*rpoD*) and six alternative σ-factors σ^54^, σ^38^, σ^32^, σ^28^, σ^24^, and σ^19^ (*rpoN*, *rpoS*, *rpoH*, *fliA*, *rpoE*, and *fecI*, respectively) have been described in *Escherichia coli*. Although the importance of σ-factors and their role in the function of the RNAP and bacterial transcription are well known, we do not yet have a genome-wide understanding of the network of regulatory interactions that the σ-factors comprise in any species. With systems biology and genome-scale science emerging and describing the phenotypic functions of bacteria, it is now possible to comprehensively elucidate the structure of the σ-factor network. Here, we present the results from a systems approach that integrates multiple genome-scale measurements to reconstruct the regulatory network of σ-factor-gene interactions in *E. coli*. This reconstruction is provided here as a resource for the scientific community.

## Results and discussion

### Determination of the genome-wide map of holoenzyme binding

To capture the first step of the transcription cycle, which is the formation of the Eσ^x^-promoter complex, we obtained genome-wide location profiles and integrated the identified RNAP and σ-factor binding sites, leading to a reconstruction of a genome-scale Eσ-binding region map (Figure 1b). A genome-wide static map of the entire group of Eσ^x^-binding sites (Eσ^x^ map) was determined by employing chromatin immunoprecipitation and microarray (ChIP-chip) of rifampicin-treated cells (Figure 1c), revealing the active promoter regions *in vivo* across the *E. coli* genome [4,5] (see Methods). A total of 2,129 Eσ^x^-binding regions were identified, consisting of 727 (34.1%) for the leading strand, 755 (35.5%) for the lagging strand, and 647 (30.4%) for both strands (that is, divergent promoter regions) (see Additional file 1: Figure S1).

**Figure 2:**
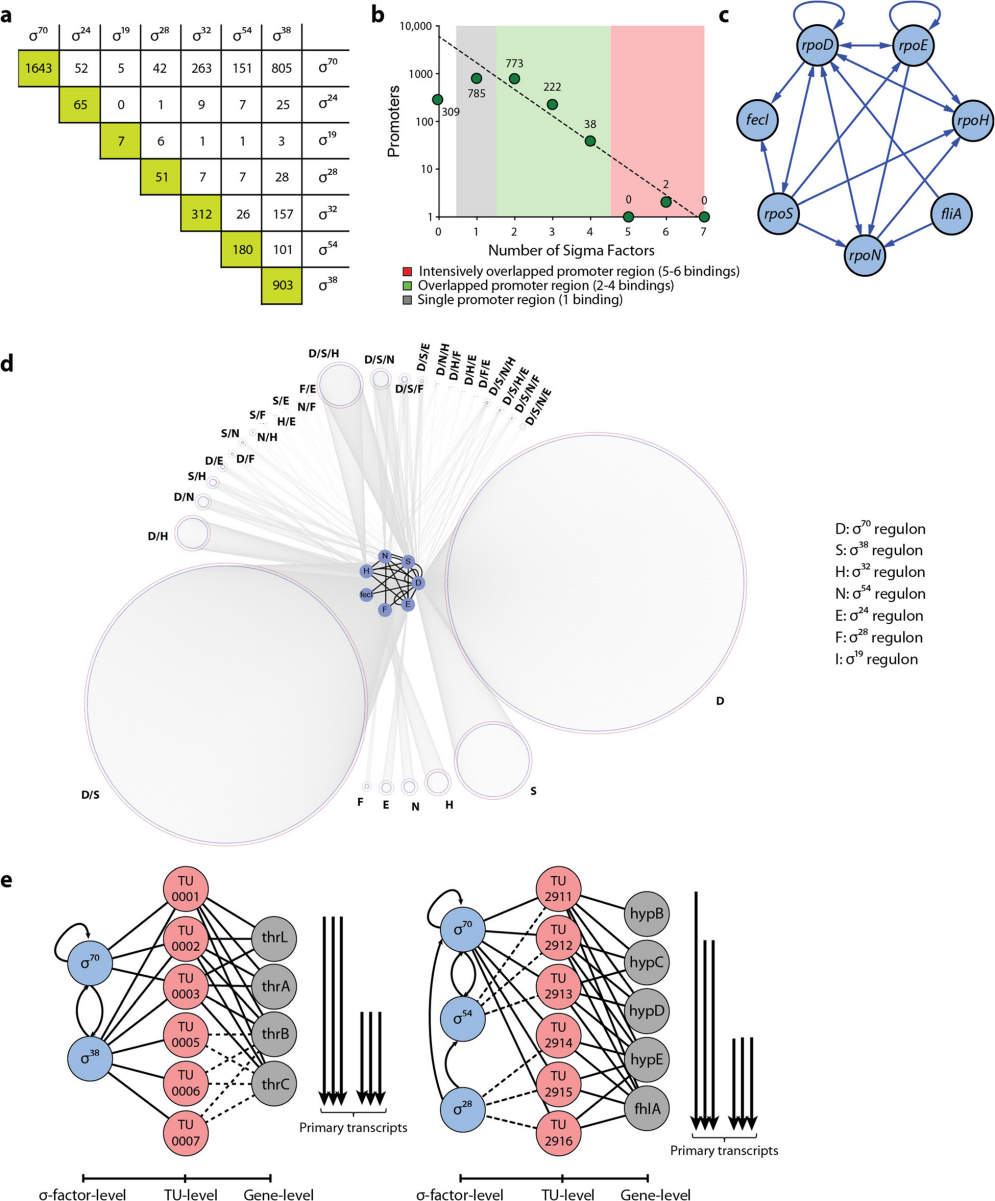
**Properties of the reconstructed σ-factor network in *Escherichia coli. *(a)** Extensive overlapping between σ-factor binding sites. For each σ-factor, σ^70^, σ^38^, σ^54^, σ^32^, σ^28^, σ^24^, and σ^19^, we identified 1,643, 903, 180, 312, 65, 51, and 7 binding regions, respectively. The number of binding regions overlapping between any two σ-factors is shown. For instance, 805 binding regions that were bound by both σ^70^ and σ^38^ were identified. **(b)** Number of promoters bound by multiple σ-factors showed a complex overlap between different σ-factors, indicating complicated alternative σ-factor usage. **(c)** A regulatory network between σ-factors in *E. coli*, in which σ^70^ and σ^38^ regulate expression of most of the seven σ-factors; σ^70^ and σ^24^ auto-regulate themselves. **(d)** Reconstruction of a three-layered network of σ-factors, transcription units (TUs), and genes. This network shows that many transcription start sites (TSSs) are shared by multiple σ-factors, suggesting possible competition between σ-factors for promoter binding. **(e)** Examples of *thrLABC* and *hypBCDE*-*fhlA* transcription units that are differently regulated by multiple σ-factors, and result in different TUs containing different sets of genes. For instance, TU001 is regulated by σ^70^ and contains four genes, *thrLABC*, while TU0005 is regulated by σ^38^ and had only two genes, *thrB* and *thrC*.

Although the construction of the Eσ^x^ map is informative, it is not sufficient to produce the σ-specific Eσ-binding map, in which the promoter-specific role of the σ-factor is detailed [6]. We thus deployed ChIP-chip assays for the direct identification of the locations of σ-factor binding across the *E. coli* genome. We analyzed *E. coli* cells grown to mid-logarithmic phase or to stationary phase under multiple growth conditions (see Additional file 2: Table S1). Using data from biological duplicate or triplicate experiments for each σ-factor ChIP-chip (36 experiments in total), we identified 1,643 targets for σ^70^, 903 targets for σ^38^, 312 targets for σ^32^, 180 targets for σ^54^, 51 targets for σ^28^, and 7 targets for σ^19^ (Figure 1c; Figure 2a; see Additional file 3: Table S2; see Additional file 4: Table S3). We were not able to obtain dataset for σ^24^, and the missing dataset was supplemented by incorporating 65 σ^24^ promoter regions from RegulonDB [6]. For validation, we compared the σ-factor binding regions with the previously reported promoters regulated by each σ-factor [6] (Figure 1d; see Additional file 5: Table S5). Overall, we identified 86% of the previously reported binding sites and 2,465 new σ-factor binding regions, extending the current knowledge by over 300% (see Additional file 5: Table S5).

By integrating the entire Eσ^x^ and σ-factor binding regions, we obtained the genome-wide Eσ-binding region map (Eσ map) comprising 3,161 binding regions (see Additional file 6: Table S4). Next, each Eσ-binding site was classified into one of three categories depending on the number of σ-factors recruited to that site: single Eσ-binding promoter region (SPR), overlapped Eσ-binding promoter region (OPR), and intensively overlapped Eσ-binding promoter region (IOPR) (Figure 1b, d; Figure 2b). For instance, all σ-factors except σ^19^ were detected at the promoter region of the *rpoD* gene, which encodes σ^70^; however, only σ^19^ was found to bind to the promoter region of the *fecABCDE* operon, which encodes the ferric citrate outer membrane receptor and the ferric citrate ATP-binding cassette (ABC) transporter (Figure 1d). Over 48% of Eσ-binding regions identified in this study were overlapped or extensively overlapped binding regions, indicating that Eσ switching, or binding of alternative Eσ, at the same promoter region may be needed to ensure continued gene expression in response to environmental changes [2] (Figure 2a).

### Determination of the genome-wide promoter map

We found that 69% of the Eσ-binding regions exhibited strand specificity, with the balance being observed as divergent promoter regions (see Additional file 1: Figure S1). Although the assignment of the RNAP-binding regions to each strand was achievable using the expression profiles [7], it was difficult to assign σ-factors directly to the promoter regions because information on the *cis*-acting sequence elements, such as the −10 and −35 boxes in the promoter regions, is not yet fully elucidated for each σ-factor. To identify the promoter elements more precisely with strand specificity and a better resolution than ChIP-chip, we performed transcription start site (TSS) profiling at the genome scale with a single nucleotide resolution. A genome-wide TSS map was generated from TSS profiling by rapid amplification of cDNA ends (RACE) followed by deep sequencing after 5′ triphosphate enrichment [8-10] for three conditions: stationary phase, heat shock, and alternative nitrogen source with glutamine. TSS profiling for exponential phase was taken from a previous study [9], and processed together with the other three datasets. The TSS map was then integrated with the Eσ map to build a strand-specific promoter map (P map) (Figure 1b-d).

### Reconstruction of sigma factor regulons and their overlaps

The P map was combined with the transcription unit (TU) map [7], resulting in the σ-factor-TU gene (σ-TUG) network (Figure 2d, e; see Additional file 7: Table S6). A network of interactions between the σ-factors was extracted from the σ-TUG network (Figure 2c). σ^70^ and σ^24^ are the only σ-factors that auto-regulate themselves, and σ^70^ and σ^38^ regulate most of the other σ-factors, reflecting their roles as housekeeping σ-factors in exponential and stationary phase [1]. Gene essentiality data are available for *E. coli*[11], and only *rpoD* has been found to be an essential σ-factor*.* This network feature is consistent with the fact that σ^70^ regulates the highest number of σ-factors, including itself. In addition, σ^70^ has the largest regulon, and this cannot be replaced by the other σ-factors (Figure 2d).

The significant overlap of σ-factor regulons leads to the fundamental questions: what is the molecular basis for the overlap, and what are the consequences of having a complicated σ-factor network? Because each σ-factor has an individual ability to recognize *cis*-acting sequence elements in the promoter region (such as −10 box or −35 box), we analyzed the sequence motifs of the promoter regions (see Additional file 1: Figure S2). As in previous studies [12-14], the sequence motifs of σ^70^ and σ^38^ were found to have a similar −10 box sequence (TAtaaT and CTAtacT); however, unlike the σ^70^ sequence motif, σ^38^ did not have a distinctive −35 box. The similarity in the −10 box sequence motifs of the σ^70^- and σ^38^-specific promoters and the degenerate nature of the −35 box sequence of the σ^38^-specific promoters explains, in part, how a large overlap between σ^70^ and σ^38^ regulons is possible.

With the structure and molecular details of the σ-TUG network in hand, we were able to study its functional states. Because of the limited number of E complexes in a growing *E. coli* cell [1], each σ-factor should compete to achieve association with an E complex to initiate transcription. Thus, it becomes important which factor Eσ^x^ binds, and how frequently it does so [15]. We found that the promoter sets specific to each σ-factor overlap extensively, and a large number of promoters bound by multiple σ-factor share the same TSS (Figure 2a,d). These findings raise questions about the molecular mechanism of σ-factor competition for binding to the E complex and subsequently to the promoter, and how that affects transcription initiation.

### Sigma factor competition in overlapped promoters

**Figure 3:**
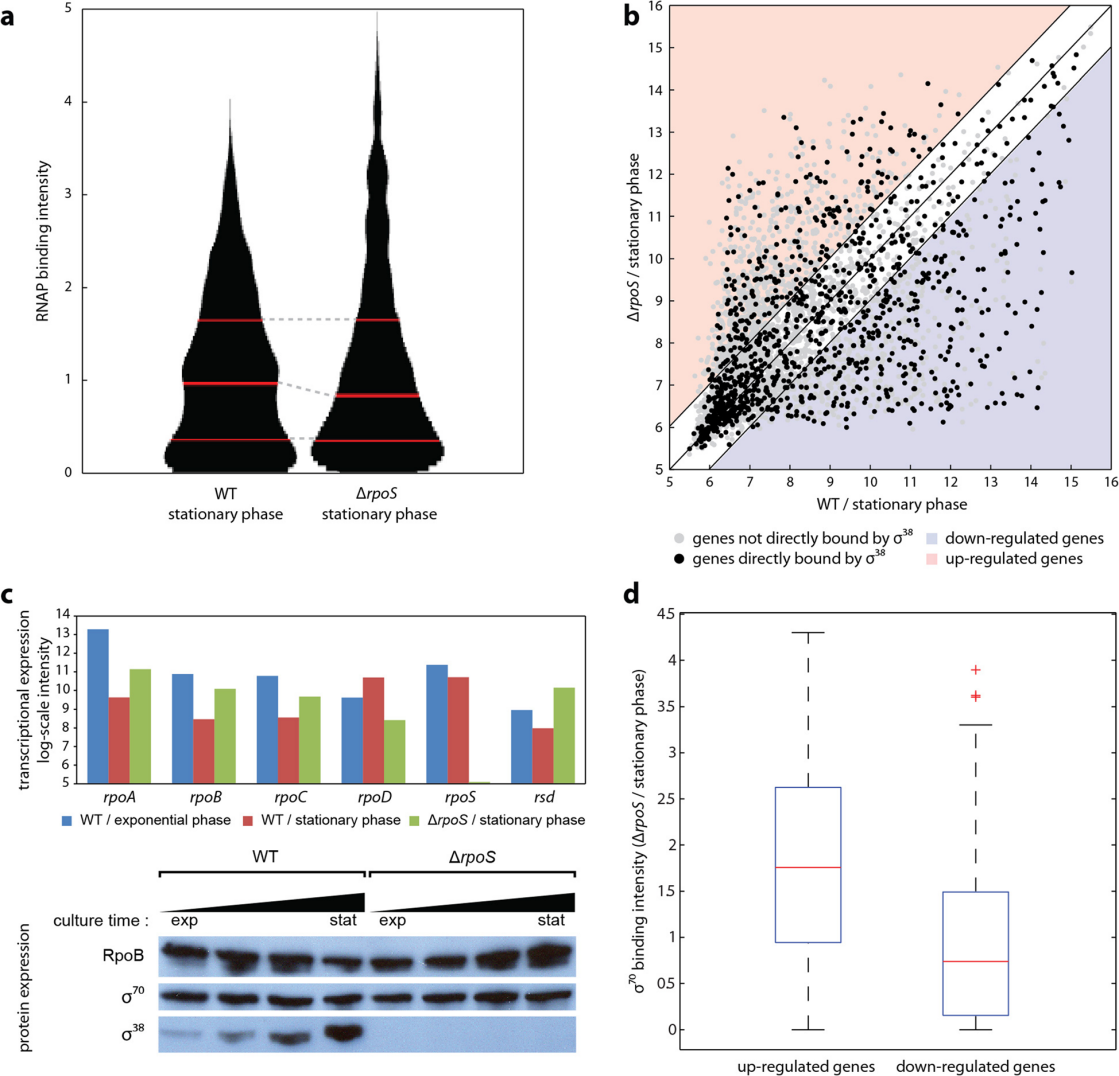
**Competition between σ**^**70 **^**and σ**^**38 **^**in overlapping promoter regions. (a)** Recruitment of RNA polymerase (RNAP) core enzyme to promoters upstream of 1,139 σ^38^-specific genes was recovered when *rpoS* was knocked out. RNAP binding intensity on the *y*-axis was the chromatin immunoprecipitation and microarray (ChIP-chip), intensity; the three red lines represent the first, second, and third quantiles. **(b)** Comparison of transcriptional expression of genes in wild type (WT) and *ΔrpoS* strains. Of 1,139 genes with σ^38^-specific promoters,178 had up-regulated transcription (red background) and 291 had down-regulated transcription (blue background). **(c)** Expression level of σ^70^ and σ^38^ was measured at both th transcriptional and translational levels. The amount of σ^70^ was abundant in exponential and stationary phase, and so it was absent in *rpoS*. **(d)** After rpoS knock-out, up-regulated genes were more strongly bound by σ^70^ than down-regulated genes.

σ-factors are believed to act predominantly as positive effectors, as they recognize the *cis*-acting elements in promoters that enable the Eσ^x^ to bind. Interestingly, however, σ^38^ has a negative effect on the expression level of some genes, even though it acts mainly as a positive effector [16,17]. To shed light on the molecular mechanisms of σ-factor competition by σ^38^, we performed ChIP-chip experiments for RpoB with wild type (WT) *E. coli* and its isogenic *rpoS* knock-out strain to obtain differential Eσ^x^ binding to the genome. The differential binding intensity of the Eσ^x^ to the promoters of 1,139 genes, whose transcription is directly affected by σ^38^, is shown in Figure 3a. If σ^38^-specific promoters were bound only by σ^38^, then the E complex recruited to those promoters would be very scarce. However, the majority of σ^38^-specific promoters showed significant levels of signaling for Eσ^x^ binding in the σ^38^ deletion strain, indicating recruitment of the Eσ^x^ and implying rescue of transcription activity (Figure 3a).

To confirm that the detected binding of the Eσ^x^ leads to transcription, we performed expression profiling with WT and *rpoS* knock-out strain cells under stationary phase conditions (Figure 3b; see Additional file 8: Table S7). Most genes having σ^38^-specific promoters were expressed. Of 1,139 genes with σ^38^-specific promoters, 178 (16%) showed up-regulated expression when *rpoS* was removed and 291 (26%) showed expression that was down-regulated more than two-fold (*t*-test *P*-value ≤0.05). The remaining 58% of the genes showed no statistical significance in expression (fold change <2) or were not expressed in either strain. In the absence of *rpoS*, σ^38^-specific promoters became active in transcription, leading to expression of the corresponding genes, but at a different level for 469 (41%) of these 1,139 genes.

Expression of genes with σ^38^-regulated genes was recovered when *rpoS* was knocked out; however, it is not known which of the other σ-factors is replacing the role of σ^38^. As σ^70^ shared the largest portion of promoters with σ^38^, it is reasonable to assume that σ^70^ would replace σ^38^ when σ^38^ is missing. In *E. coli* MC4100, it was reported that the amount of σ^70^ is in abundance during stationary phase [18]. Similarly, we found that *E. coli* K-12 MG1655 also showed high protein expression of σ^70^ during stationary phase in WT and *ΔrpoS* strain (Figure 3c, see Additional file 1 for detailed description). In addition, we examined how many genes bound by σ^38^ in the WT strain were bound by σ^70^ when *rpoS* was deleted. We found that about 89% of those genes was bound by σ^70^ when σ^38^ was missing, (see Additional file 1: Figure S3). This unexpectedly high rate of σ-factor substitution explains how the majority of genes directly bound by σ^38^ recovered their expression when *rpoS* was knocked out (Figure 3b). However, it is still unclear how some of those genes were up-regulated.

Because approximately 89% of these genes were bound by σ^70^, we measured the intensity of σ^70^ binding in Δ*rpoS* during stationary phase with ChIP-chip experiments, and compared the binding intensity between up-regulated and down-regulated genes (Figure 3d; see Additional file 1: Figure S4). This measurement showed that up-regulated genes were bound more strongly by σ^70^ (*P*-value of Wilcoxon rank sum test was 4.80 × 10^-18^), suggesting that strong σ^70^ binding resulted in increased transcription. This finding indicates that the presence of σ^38^ actually contributed to repressing the transcriptional expression of some genes, presumably by competition for shared promoters between σ^70^ and σ^38^.

### Comparative analysis of the sigma factor network in closely related species

With the detailed reconstruction of the σ-TUG network in *E. coli*, we could now address the issue of the difference between such networks in closely related species. Genome-wide identification of TSSs of two gamma-Proteobacteria, *E. coli* and *Klebsiella pneumoniae*, revealed that promoter regions upstream of orthologous genes are differently organized in the two species, resulting in different usage of TSSs [9]. As σ-factors recognize sequence elements of promoters, and they are directly upstream of TSSs, it is important to determine any differences in σ-factor binding patterns. Whereas the *E. coli* genome contains seven σ-factors, *K. pneumoniae* is known to have only five, missing *fliA* and *fecI*, which are found in *E. coli*. The five σ-factors that the two species have in common are highly conserved in terms of amino acid sequence similarity: 95.9% for *rpoD*, 98.5% for *rpoS*, 89.8% for *rpoN*, 95.1% for *rpoH*, and 96.3% for *rpoE*. Promoter sequence motifs examined from the TSSs were found to be identical between *E. coli* and *K. pneumoniae*, suggesting that the sequence motifs for each orthologous σ-factor are identical [9,19]. However, the different organization of upstream regulatory regions of the two species and the different pattern of transcription initiation indicates the possibility of significantly diverse σ-factor binding.

**Figure 4:**
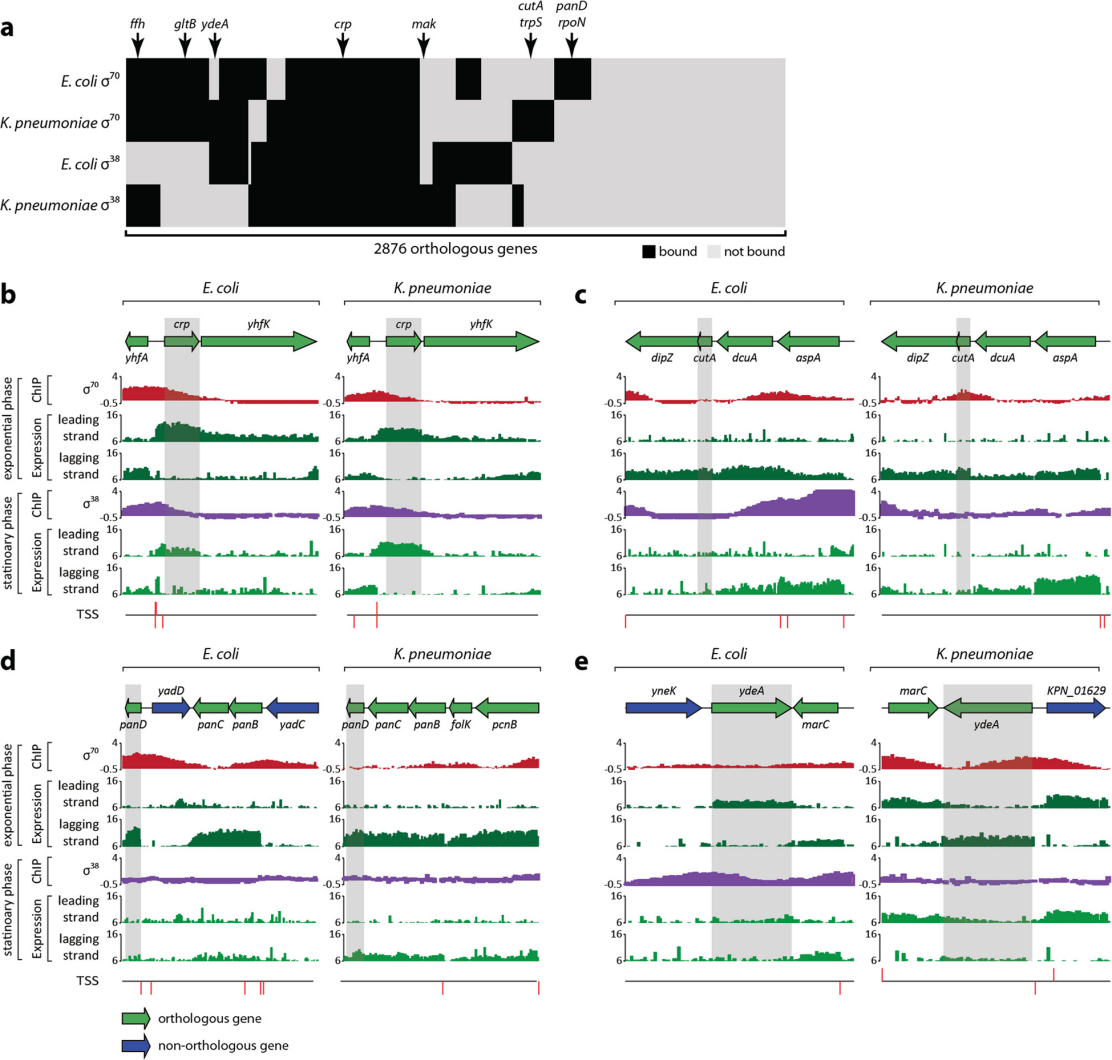
**Conservation and divergence in transcriptional regulation by σ-factors. (a)** Clustering σ-factor binding patterns revealed conserved and divergent transcriptional regulation of 2,876 orthologous genes. **(b)***crp* is regulated by σ^70^ and σ^38^ in both species, showing regulation conservation. **(c)** In *Esherichia coli*, *cutA* is a part of the *dcuA-cutA-dipZ* transcription unit (TU) and is regulated by σ^70^ and σ^38^, while *cutA* in *Klebsiella pneumoniae* is the first gene in its TU, and is directly bound by σ^70^. **(d)** In *K. pneumoniae*, *panD* is a part of the *panBCD* TU, which is regulated by σ^70^. However, in *E. coli*, *panD* is separated from *panBC* by *yadD*, making another distinct TU. These two TUs are both regulated by σ^70^. **(e)** A genomic region containing *ydeA* and *marC* in both species was inverted, and this genomic inversion was accompanied by a transcription regulation switch between σ^70^ and σ^38^.

To investigate the binding patterns of two major σ-factors, *rpoD* and *rpoS*, we analyzed ChIP-chip datasets for σ^70^ under exponential phase and σ^38^ under stationary phase grown in glucose minimal media as described previously [19]. *E. coli* and *K. pneumoniae* have 4,513 and 5,305 genes, respectively, and 2,876 coding genes were defined as orthologs by two-way reciprocal alignment. Binding of σ^70^ and σ^38^ under specified conditions upstream of those orthologous genes was analyzed and clustered (Figure 4a). Of the 2,876 orthologous genes, 60% showed the same binding patterns (584 had both σ factors bound, 213 had σ^70^ bound, 102 had σ^38^ bound, and 847 had neither factor bound). These two closely related bacteria, *E. coli* and *K. pneumoniae,* share the majority of their gene contents, with most of the open reading frames having highly conserved sequences. However, conserved genes showed significantly different σ-factor binding patterns, indicating diverse gene regulation by different transcription initiation (Figure 4c,d). Interestingly, in some cases, altered binding of σ-factors was associated with changes in TU organization, suggesting even more diverse regulation between the two species. Although two major σ-factors were found to bind differently upstream of orthologous genes, regulation between σ-factors remained unchanged, except for the two missing σ-factors, *fliA* and *fecI*, in *K. pneumoniae* (see Additional file 1: Figure S5). Thus, regulation of gene expression by σ-factors may evolve faster than regulation among the σ-factors themselves.

## Conclusions

Genome-scale measurements enabled us to reconstruct the σ-TUG network in *E. coli* K-12 MG1655. This network is at the core of transcriptional regulation in bacteria. Its reconstruction has enabled the assessment of its topological characteristics, functional states, and limited comparison with related species. With the integration of a growing body of experimental data on transcription factor (TF) binding and activity, the resource provided here opens up the possibility of developing a comprehensive reconstruction of the entire transcriptional regulatory network in *E. coli*, which would simultaneously describe the function of σ-factors and TFs that produce the entire expression state of the organism.

## Methods

### Bacterial strains, media, and growth conditions

*E. coli* K-12 MG1655 and its isogenic knock-out strains were used in this study. The deletion mutants (*ΔrpoS and ΔrpoN)* were generated by a λ Red and FLP-mediated site-specific recombination system [20]. *E. coli* cells were harvested at mid-exponential phase (optical density at 600 nm (OD_600nm_) of approximately 0.5) with the exception of stationary phase experiments (OD_600nm_ approximately 1.5). Glycerol stocks of *E. coli* strains were inoculated into M9 or W2 minimal media [21] (for nitrogen-limiting condition) with glucose (2 g/l) and cultured overnight at 37°C with constant agitation. Cultures were then diluted 1:100 into 50 ml of fresh minimal media. and cultured at 37°C to appropriate cell density. For heat-shock experiments, cells were grown to mid-exponential phase at 37°C. and half of the culture was used as a control, while the remaining culture was transferred into pre-warmed (50°C) media and incubated for 10 minutes. For nitrogen-limiting condition, ammonium chloride in the minimal media was replaced by glutamine (2 g/l).

### Total RNA isolation

Cell (3 ml) culture was mixed with 6 ml RNAprotect Bacteria Reagent (Qiagen, Valencia, CA, USA). Samples were mixed immediately by vortexing for 5 seconds, incubated for 5 minutes at room temperature, then centrifuged at 5000 × *g* for 10 minutes. The supernatant was decanted, and any residual supernatant was removed by inverting the tube once onto a paper towel. Total RNA samples were then isolated using an RNeasy Plus Mini Kit (Qiagen) in accordance with the manufacturer’s instructions. Samples were then quantified using a NanoDrop 1000 spectrophotometer (Thermo Scientific), and the quality of the isolated RNA was checked by visualization on agarose gels and by measuring the ratio of the absorbance at 260 and 280 nm (A_260_/A_280_ ratio) of the sample (>1.8).

### Transcriptome analysis

Transcriptome datasets with oligonucleotide tiling microarrays for WT *E. coli* K-12 MG1655 grown under four conditions (exponential phase, stationary phase, heat shock, and nitrogen-limiting condition), were taken from a previous study [7]. In order to obtain a transcriptome dataset for *E. coli* deletion mutant *ΔrpoS*, a previously described protocol [9] was adapted for the deletion mutant in the current study. Briefly, 10 μg of purified total RNA sample was reverse transcribed to cDNA with amino-allyl dUTP. The amino-allyl-labeled cDNA samples were then coupled with Cy3 monoreactive dyes (Amersham). Cy3-labeled cDNAs were fragmented to the 50 to 300 bp range with DNase I (Epicentre). High-density oligonucleotide tiling arrays consisting of 371,034 50-mer probes spaced 25 bp apart across the whole *E. coli* genome were used (Roche Nimblegen). Hybridization, washing, and scanning were performed in accordance with the manufacturer’s instructions. Three biological replicates were used for stationary phase in glucose minimal media. Probe level data were normalized with a robust multiarray analysis (RMA) algorithm without background correction, as implemented in NimbleScan 2.4 software.

### TSS-sequencing by modified 5′ RACE, and deep sequencing

The raw TSS dataset for exponential phase was taken from a previous study [9]. For the other three conditions (stationary phase, heat shock, and nitrogen-limiting condition), the previously described TSS determination protocol [9] was adapted for *E. coli* K-12 MG1655. To enrich intact 5′ tri-phosphorylated mRNAs from the total RNA, 5′ mono-phosphorylated rRNA and any degraded mRNA were removed by treatment with a Terminator 5′-Phosphate Dependent Exonuclease (Epicentre) at 30°C for 1 hour. The reaction mixture consisted of 10 μg purified total RNA, 1 μl terminator exonuclease, reaction buffer, and RNase-free water up to total 20 μl. The reaction was terminated by adding 1 μl of 100 mM EDTA (pH 8.0). Intact tri-phosphorylated RNAs were precipitated by adding 1/10 volume of 3 M sodium acetate (pH 5.2), 3 volumes of ethanol, and 2 μl of 20 mg/ml glycogen. RNA was precipitated at −80°C for 20 minutes and pelleted, washed with 70% ethanol, dried in Speed-Vac for 7 minutes without heat, and resuspended in 20 μl nuclease free water. The tri-phosphorylated RNA was then treated with RNA 5′-Polyphosphatase (Epicentre) to generate 5′-end mono-phosphorylated RNA for adaptor ligation. The RNA sample from the previous step was mixed with 2 μl 10× reaction buffer, 0.5 μl SUPERase-In (Ambion), 1 μl RNA 5′-Polyphosphatase, and RNase-free water up to 20 μl. The mixture was incubated at 37°C for 30 minutes and reaction was stopped by phenol-chloroform extraction. Ethanol precipitation was carried out for isolating the RNA as described above. To ligate the 5′ small RNA adaptor (Table 1) to the 5′-end of the mono-phosphorylated RNA, the enriched RNA samples were incubated with 100 μM of the adaptor and 2.5 U of T4 RNA ligase (New England Biolabs). cDNAs were synthesized using the adaptor-ligated mRNAs as template using a modified small RNA RT primer from Illumina (Table 1) and Superscript II Reverse Transcriptase (Invitrogen). The RNA was mixed with 25 μM modified small RNA RT primer and incubated at 70°C for 10 minutes and then at 25°C for 10 minutes. RT was carried out at 25°C for 10 minutes, 37°C for 60 minutes, and 42°C for 60 minutes, followed by incubation at 70°C for 10 minutes. The RT reaction mixture consisted of 5× first^t^ strand buffer; 0.01 M DTT, 10 mM dNTP mix, 30 U SUPERase•In (Ambion), and 1500 U SuperScript II (Invitrogen). After the reaction, RNA was hydrolyzed by adding 20 μl of 1 N NaOH and incubating the mixture at 65°C for 30 minutes. The reaction mixture was neutralized by adding 20 μl of 1 N HCl. The cDNA samples were amplified using a mixture of 1 μl cDNA, 10 μl Phusion HF buffer (NEB), 1 μl dNTPs (10 mM), 1 μl SYBR Green (Qiagen), 0.5 μl HotStart Phusion (NEB), and 5 pM small RNA PCR primer mix. The amplification primers used are shown in Table 1. The PCR mixture was denatured at 98°C for 30 seconds and cycled to 98°C for 10 seconds, 57°C for 20 seconds, and 72°C for 20 seconds. Amplification was monitored by a LightCycler (Bio-Rad) and stopped at the beginning of the saturation point. Amplified DNA was run on a 6% Tris-borate-EDTA (TBE) gel (Invitrogen) by electrophoresis, and DNA fragments ranging from 100 to 300 bp were size-fractionated. Gel slices were dissolved in two volumes of EB buffer (Qiagen) and 1/10 volume of 3 M sodium acetate (pH 5.2). The amplified DNA was then ethanol-precipitated and resuspended in 15 μl DNase-free water (USB). The final samples were then quantified using a NanoDrop 1000 spectrophotometer (Thermo Scientific).

**Table 1:** Primers used in the study

**Primer**	**Direction**	**Sequence 5′→3′**
Small RNA adaptor	-	GUUCAGAGUUCUACAGUCCGACGAUC
Small RT primer	-	CAAGCAGAAGACGGCATACGANNNNNNNNN
Amplification primers	Forward	AATGATACGGCGACCACCGACAGGTTCAGAGTTCTACAGTCCGA
	Reverse	CAAGCAGAAGACGGCATACGA

RT, reverse transcription.

### Sequencing, data processing, and mapping

The data processing and mapping of the sequencing results to obtain potential TSSs was performed exactly as described previously [9]. In brief, the amplified cDNA libraries from two biological replicates for each condition were sequenced on an Illumina Genome Analyzer. Sequence reads for cDNA libraries were aligned to the *E. coli* K-12 MG1655 genome (NC_000913) using Mosaik [22] with the following arguments: hash size = 10, mismatach = 0, and alignment candidate threshold = 30 bp. Only reads that aligned to a unique genomic location were retained. Two biological replicates were processed separately, and only sequence reads presented in both biological replicates were considered for further processing. The genome coordinates of the 5′-end of these uniquely aligned reads were defined as potential TSSs, and of these, only TSSs with the strongest signal within 10 bp window were kept, in order to remove possible noise signals. TSSs with signals that were 40% or greater of the strongest signal upstream of an annotated gene were considered as multiple TSSs. The strongest signal was defined as the potential TSS that had the highest number of reads out of all the TSSs upstream of an annotated gene. For further analysis, TSSs lying within RNAP-binding regions (see Additional file 4: Table S3) were used for integration with σ-factor binding information.

### Chromatin immunoprecipitation and microarray analysis

Briefly, the immunoprecipitated RNAP-associated DNA fragments were fluorescently labeled and hybridized to a high-density oligonucleotide tiling microarray representing the entire *E. coli* genome [5]. To identify *in vivo* binding regions of RNAP complex and six σ-factors (σ^70^, σ^54^, σ^38^, σ^32^, σ^28^, and σ^19^), we isolated DNA fragments bound to those RNAP subunits from formaldehyde-crosslinked *E. coli* cells, using ChIP with six different antibodies that specifically recognize each subunit (NeoClone). An *E. coli* strain harboring RpoH-8myc was constructed as previously described [23,24], and used for the σ^38^ ChIP-chip with anti-c-myc antibody (9E10; Santa Cruz Biotechnologies). Cells were grown under appropriate conditions (see Additional file 2: Table S1) and harvested. The immunoprecipitation (IP) DNA and mock-IP DNA were hybridized onto high-resolution whole-genome tiling microarrays, which contained a total of 371,034 oligonucleotides with 50-bp probes overlapping by 25 bp on both forward and reverse strands. Tiling microarrays were hybridized, washed, and scanned in accordance with the manufacturer’s instructions (Roche NimbleGen). To increase the depth of the number of promoter regions identified, datasets were generated under multiple growth conditions with a total number of 45 ChIP-chip experiments (36 for σ-factors and 9 for RNAP), and analyzed (see Additional file 2: Table S1). We were not able to obtain results for the ChIP-chip experiment for σ^24^. This could be because the expression level of σ^24^ was not high enough, or the conditions were not appropriate to activate σ^24^. To remedy the missing dataset, we deployed known binding information for σ^24^ from the public database [25].

### ChIP-chip data analysis

The analysis was performed, as previously described [7,26]. In brief, TF-binding regions were identified by using the peak-finding algorithm built into the NimbleScan software (Roche NimbleGen). Processing of ChIP-chip data was performed in three steps: normalization, IP/mock-IP ratio computation (in log_2_ scale) and enriched-region identification. The log_2_ ratios of each spot in the microarray were calculated from the raw signals obtained from both Cy5 and Cy3 channels, and then the values were scaled by Tukey bi-weight mean. The log_2_ ratio of Cy5 (IP DNA) to Cy3 (mock-IP DNA) for each point was calculated from the signals, then, the bi-weight mean of this log_2_ ratio was subtracted from each point. Each log-ratio dataset (from duplicate or triplicate samples) was used to identify TF-binding regions using the software (width of sliding window = 300 bp). Our approach to identify the TF-binding regions was to first determine the binding locations from each dataset, and then combine the binding locations from at least five of six datasets to define a binding region, using the recently developed MetaScope visualization software and genome browser [27].

### Western blotting

*E. coli* K-12 MG1655 and Δ*rpoS* deletion mutant cells were grown in M9 minimal media with 0.2% glucose, and were harvested from mid-exponential phase to stationary phase every 2 hours. Cells were pelleted by centrifugation, and were lysed with lysozyme in a lysis buffer (10 mM Tris–HCl (pH 7.5), 100 mM NaCl, and 1 mM EDTA. The supernatant was decanted after centrifugation to remove unlysed cells. The concentration of total protein in the lysate was measured with Qubit Protein Assay Kit (invitrogen), and 5 μg of total protein sample were mixed with 4× SDS-PAGE sample loading buffer (Invitrogen) and 10 mM DTT, then boiled at 90°C for 5 minutes. The boiled samples were separated by electrophoresis with 10% Bis-Tris gel in MOPS buffer, and transferred onto Hybond-ECL membrane (Amersham Biosciences). The membrane was briefly washed in TBS with 0.1% Tween-20 (1× TBS-T) for 5 minutes on a rocker, and then treated with 2% skim milk in TBS-T buffer for 1 hour with gentle shaking. The membrane was washed twice with TBS-T for 5 minutes each on a rocker, and then it was sliced into three pieces. with RpoB, σ^70^, and σ^38^ in each slice. Sliced membranes were treated with anti-RpoB, anti-σ^70^, and anti-σ^38^ antibodies (1:10,000 dilution; NeoClone) for 1 hour on a rocker. The membrane slices were washed once in TBS-T for 15 minutes, followed by three washes of 5 minutes each, and then treated with HRP-conjugated anti-mouse IgG (1:10,000 dilution; Amersham Bioscience) in dilution for 30 minutes on a rocker, followed by one wash in TBS-T for 15 minutes and three washes of 5 minutes each. Chemiluminescent detection was applied to peroxidase conjugates on membrane to detect the amount of RpoB, σ^70^, and σ^38^.

### Availability of supporting data

All raw and processed data files have been deposited to Gene Expression Omnibus (accession number GSE46740).

## Abbreviations

ABC: ATP-binding cassette; ChIP: Chromatin immunoprecipitation; ChIP-chip: chromatin immunoprecipitation and microarray; E: RNA polymerase core enzyme; IOPR: Intensively overlapped Eσ-binding promoter region; IP: immunoprecipitation; OPR: Overlapped Eσ-binding promoter region; RACE: rapid amplification of cDNA ends; RMA: Robust multiarray analysis; RNAP: RNA polymerase; SPR: Single Eσ-binding promoter region; TBE: Tris-borate-EDTA; TF: transcription factor; TSS: Transcription start site; TU: Transcription unit; σ-TUG: σ-factor-transcription unit gene; WT: wild type.

## Competing interests

The authors declare that they have no competing interests.

## Authors’ contributions

BKC, DK, and BOP conceived the idea and designed the research. BKC, DK, and EMK performed the experiments. D, and BKC analyzed the data. DK, BKC, KZ, and BOP wrote the paper, with comments from other authors. All of the authors read and approved the final manuscript.

## Supplementary Material

Additional file 1: Figure S1 Strand specificity of RNA polymerase (RNAP) binding. **Figure S2.** Sequence motifs of σ-factors. **Figure S3.** The majority of σ^38^-specific promoters were bound by σ^70^ when *rpoS* is missing. **Figure S4.** Examples of up-regulated and down-regulated genes when *rpoS* was knocked out. **Figure S5.** Comparison of transcriptional regulation by two major σ-factors, σ^70^ and σ^38^, in two closely related bacteria. **Figure S6.** Comparison of transcriptional level of σ-factors and their anti-σ-factors. **Figure S7.** Purine and pyrimidine preferences at transcription start site (TSS) and −1 site. **Figure S8.** Number of TSSs found in one or multiple conditions. **Figure S9.** Clusters of Orthologous Groups (COG) clustering analysis of σ-factor regulons. Click here for file

Additional file 2: Table S1 *Escherichia coli* strains and culture conditions for chromatin immunoprecipitation and microarray (ChIP-chip) experiments. Click here for file

Additional file 3: Table S2 RRNA polymerase (RNAP) and σ-factor binding regions in *Escherichia coli.* Click here for file

Additional file 4: Table S3 Binding intensities of RNA polymerase (RNAP) and σ-factor binding regions. Click here for file

Additional file 5: Table S5 Comparison of σ-factor binding regions with known binding regions. Click here for file

Additional file 6: Table S4 Identified TSSs of *Escherichia coli* under four different conditions. Click here for file

Additional file 7: Table S6 Reconstructed σ-factor-transcription unit gene (σ-TUG) network in *Escherichia coli.* Click here for file

Additional file 8: Table S7 Transcription levels of *Escherichia coli* genes in the wild type and the Δ*rpoS* strain. Click here for file
