# Supplementary material for: Genome-scale reconstruction of the sigma factor network in Escherichia coli: topology and functional states
Source: BMC Biol. 2014 Jan 24;12:4. doi: 10.1186/1741-7007-12-4 (PMC3923258; doi:10.1186/1741-7007-12-4)
Supplement: Additional file 1: Figure S1 — Strand specificity of RNA polymerase (RNAP) binding. Figure S2. Sequence motifs of σ-factors. Figure S3. The majority of σ38-specific promoters were bound by σ70 when rpoS is missing. Figure S4. Examples of up-regulated and down-regulated genes when rpoS was knocked out. Figure S5. Comparison of transcriptional regulation by two major σ-factors, σ70 and σ38, in two closely related bacteria. Figure S6. Comparison of transcriptional level of σ-factors and their anti-σ-factors. Figure S7. Purine and pyrimidine preferences at transcription start site (TSS) and −1 site. Figure S8. Number of TSSs found in one or multiple conditions. Figure S9. Clusters of Orthologous Groups (COG) clustering analysis of σ-factor regulons. [file 1741-7007-12-4-S1.docx]

**Supplemental Information**

**SUPPLEMENAL FIGURES**

**
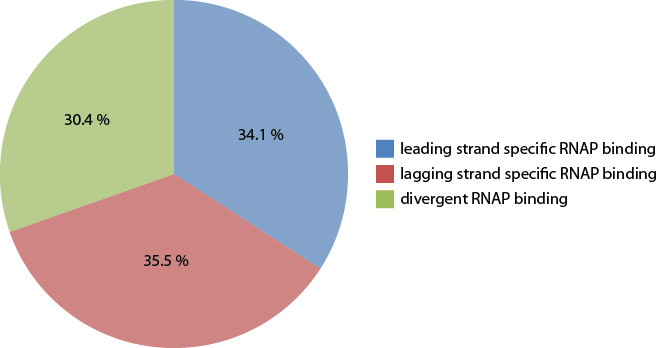
**

**Figure S1. Strand specificity of RNAP bindings.**


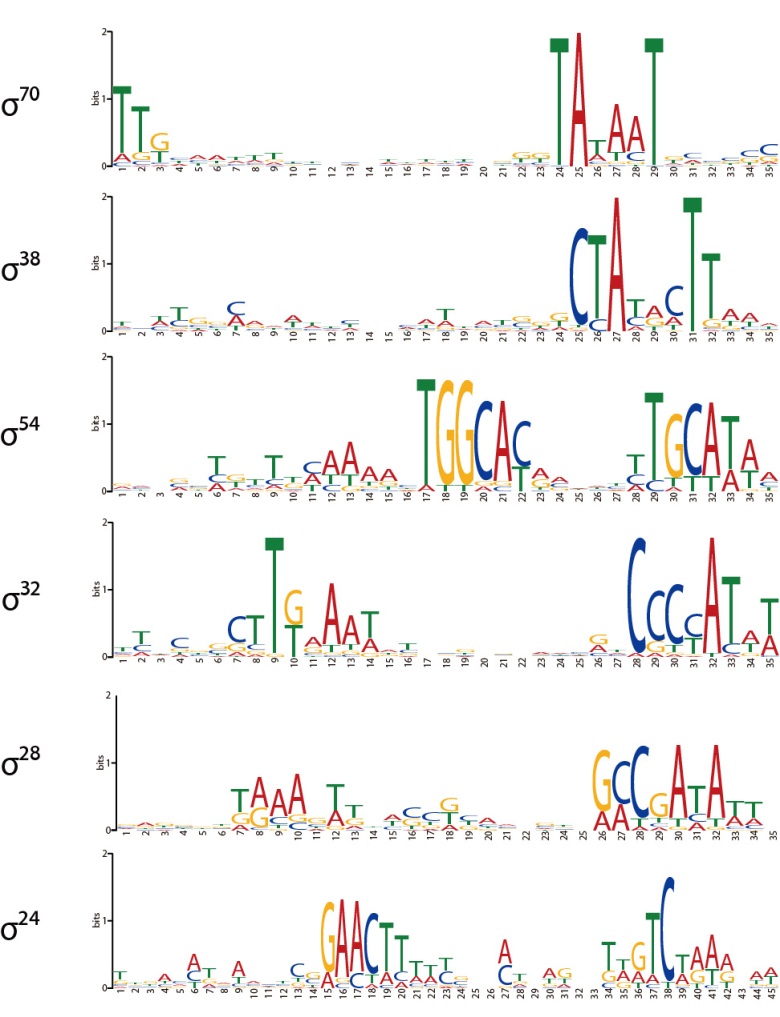


**Figure S2. Sequence motifs of σ-factors.**


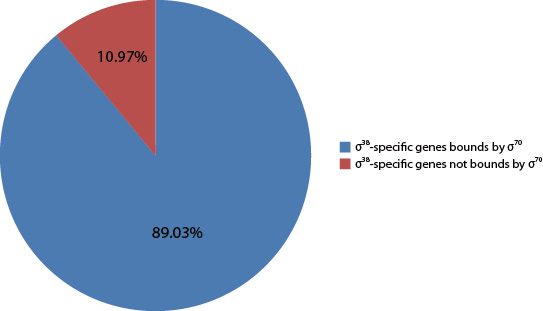


**Figure S3. The majority of σ^38^-specific promoters were bound by σ^70^ when *rpoS* is missing.**


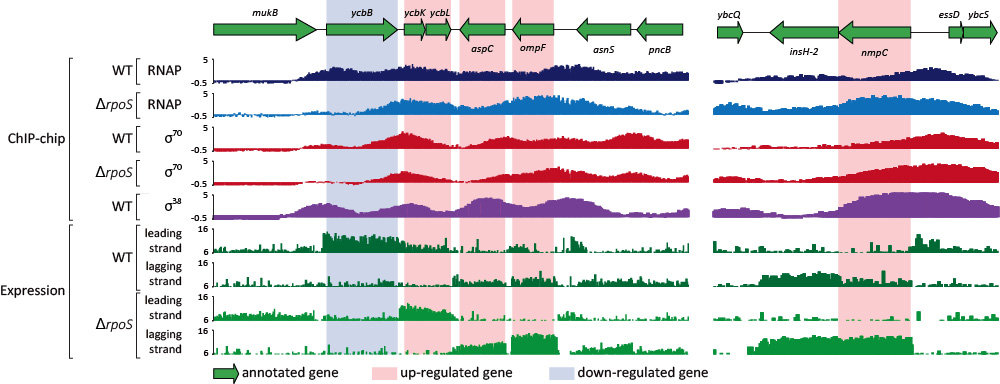


**Figure S4. Examples of up-regulated and down-regulated genes when *rpoS* is knocked out.** *ycbB* is an example of a down-regulated gene upon *rpoS* knock-out, and *ycbK*, *ycbL,* and *nmpC* are up-regulated genes. *ycbB* was not bound by σ^70^ when σ^38^ was missing, which resulted in no significant recruitment of RNAP enzyme complex, which was supported by no transcriptional expression of the particular gene. On the other hand, *nmpC* was more strongly bound by σ^70^ when σ^38^ was absent, which resulted in more RNAP binding and stronger expression.


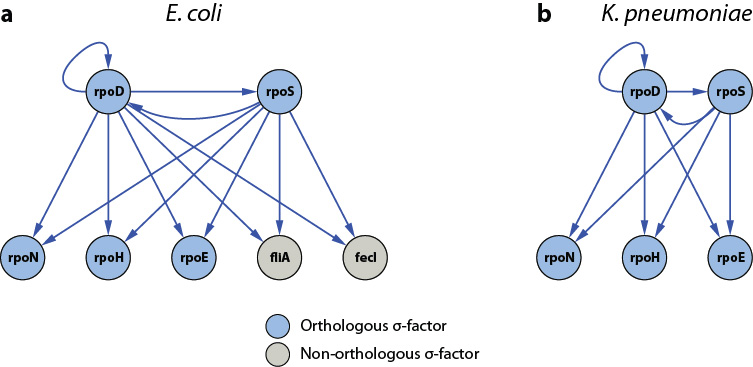


**Figure S5. Comparison of transcriptional regulation by two major σ-factors, σ^70^ and σ^38^, in two closely-related bacteria.**


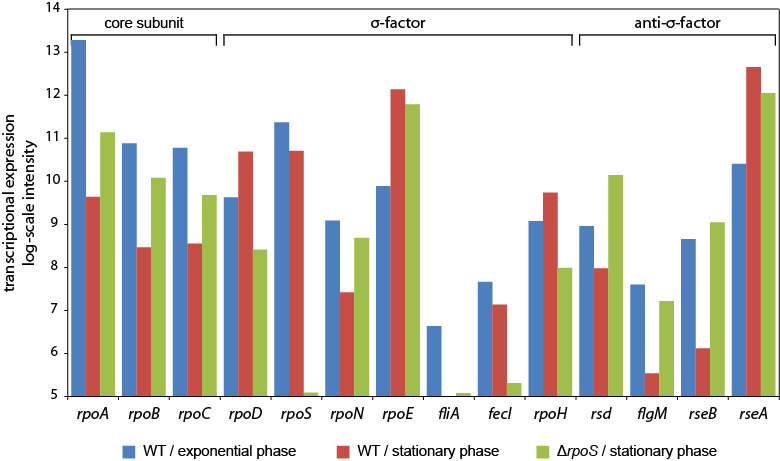


**Figure S6. Comparison of transcriptional level of σ-factors and their anti-σ-factors.**


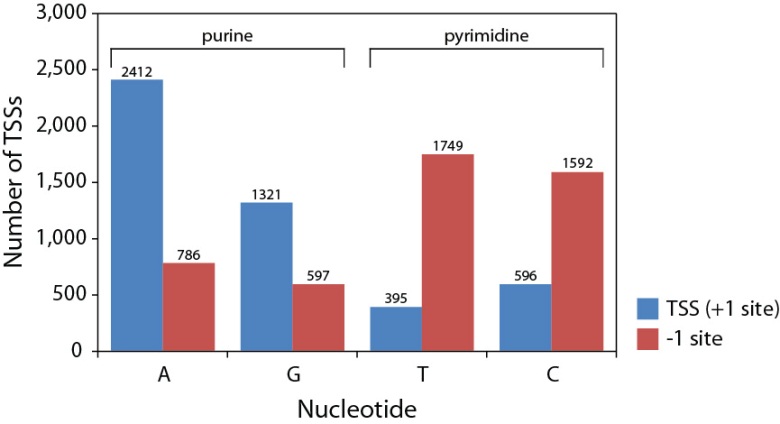


**Figure S7. Purine and pyrimidine preferences at TSS and -1 site.**


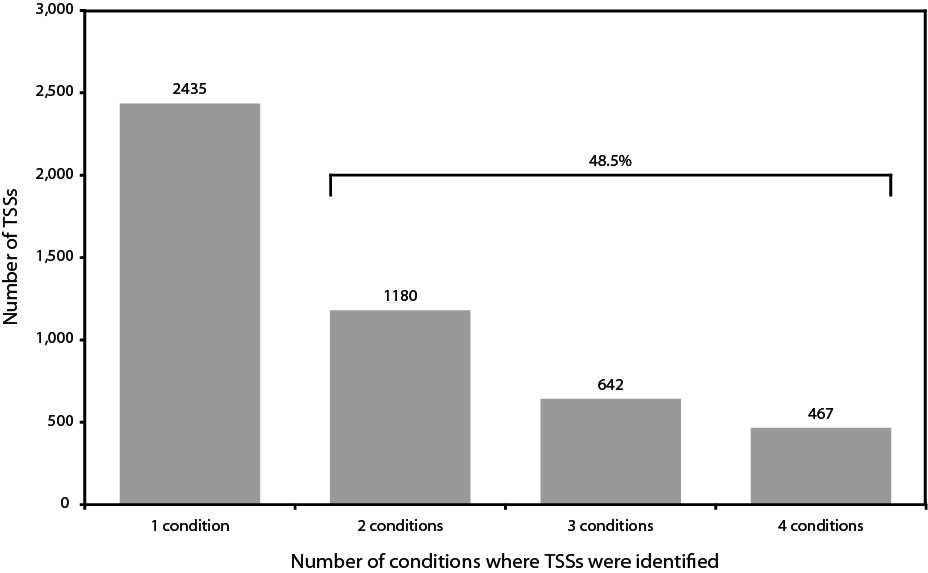


**Figure S8. Number of TSSs found in one or multiple conditions.**

**
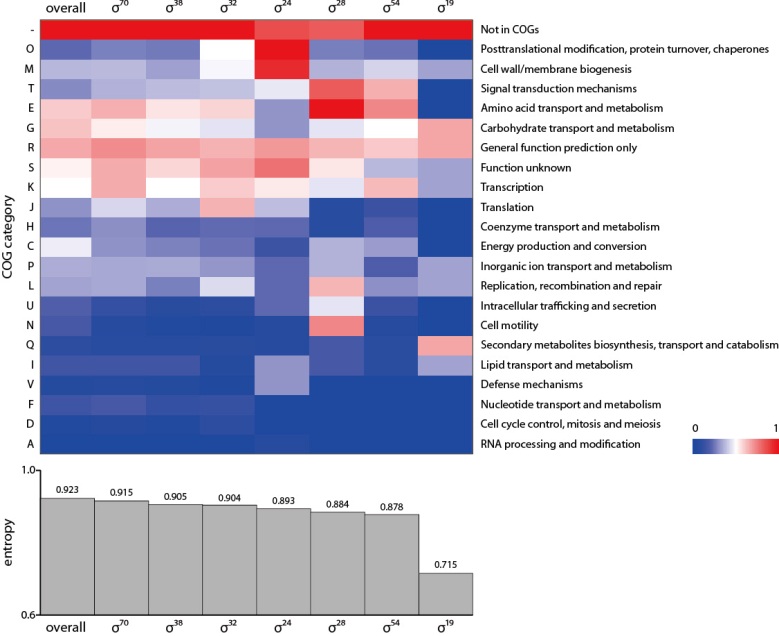
**

**Figure S9. COG clustering analysis of σ-factor regulons.**

**SUPPLEMENTAL TABLES**

**Table S1.** *E. coli* strains and culture conditions for ChIP-chip experiments

**Table S2.** RNAP and σ-factor binding regions in *E. coli*

**Table S3.** Binding intensities of RNAP and σ-factor binding regions

**Table S4.** Identified TSSs of *E. coli* under 4 different conditions

**Table S5.** Comparison of σ-factor binding regions with known binding regions

**Table S6.** Reconstructed σ- TUG network in *E. coli*

**Table S7.** Transcription levels of *E. coli* genes in the wild type and *ΔrpoS* strain

**SUPPLEMENTAL NOTES**

**Previously reported negative regulation by σ^38^**

The up-regulation of genes with σ^38^-specific promoters when *rpoS* is deleted is consistent with previous studies [[1](#_ENREF_1), [2](#_ENREF_2)] investigating negative regulation by σ^38^. *uspA* and *uspB*, encoding universal stress proteins, are divergently transcribed stasis-induced genes. The expression of *uspA* that was thought to have a σ^70^-specific promoter increased when *rpoS* was mutated, while the expression of *uspB* with a σ^38^-specific promoter was down-regulated[[1](#_ENREF_1)]. The expression data from this study confirmed this expression pattern. In another independent study[[2](#_ENREF_2)], 7 other genes, *fimA*, *glnQ*, *mglA*, *mutH*, *mutS*, *sdhA,* and *xylF*, were found as negatively affected by σ^38^, and they all agreed with our data. Thus, negative regulation by presence of σ^38^ is a regulation mechanism that is not limited to a small number of genes, but covers ~4% of the whole genome.

**Expression level of σ^70^ during stationary phase**

To confirm the expression level of transcripts and proteins in the *E. coli* K-12 MG1655 strain, we analyzed the transcriptomic expression level of genes encoding RNAP core subunits, σ-factors, and anti-σ factors under 3 different conditions: WT during exponential phase, WT during stationary phase, and Δ*rpoS* during stationary phase (**Figure 3c, Figure S5**). Compared to exponential phase, the transcription level of all RNAP core subunits, *rpoA*, *rpoB*, *rpoC* decreased significantly during stationary phase, whereas transcription of *rpoD* actually increased, and *rsd* transcription was down-regulated. This observation suggests the possibility of the concentration of σ^70^ being high enough to have a significant role during stationary phase. Unlike *rpoD*, the transcription change of *rpoS* between exponential and stationary phase was less dramatic, resulting in a slight decrease (**Figure 3c**). However both *rpoD* and *rpoS* mRNA transcripts are subject to complicated post-transcriptional regulation in *E. coli[*[*2*](#_ENREF_2)*]*, necessitating measurement of protein levels of those gene products. In *E. coli* WT, decrease of protein level of RpoB was accompanied with decrease of their transcriptional level, whereas the protein level of σ^70^ remained similar, and the amount of σ^38^ increased as cells entered stationary phase (**Figure 3c**). Thus, in *E. coli* K-12 MG1655, the expression level of σ^70^ in stationary phase is as high as in exponential phase, while the expression level of σ^38^ increases as cells enter stationary phase.

Does the expression level of σ^70^ change in in Δ*rpoS* strain during stationary phase? The answer to this question is important because σ^38^ binds directly upstream of *rpoD* and regulates its transcription (**Figure 1d**, **Figure 3c**). Based on the transcriptomic data, the transcriptional expression of *rpoD* indeed decreased by 4.9 fold, however the translational expression of σ^70^ did not decrease upon *rpoS* knock-out (**Figure 3c**). Thus, in conclusion, σ^70^ is the σ-factor with the highest concentration under stationary phase, and this is also the case when *rpoS* is knocked out, although some fraction of σ^70^ may be bound to Rsd, an anti-σ^70^ factor.

**Comparison of σ-factor binding between *E. coli* and *K. pneumoniae***

*crp* provides a good example for conserved σ-factor binding (**Figure 4c**). In both species, *crp* was bound by σ^70^ and σ^38^ in exponential and stationary phase, and the transcription initiation was detected by TSSs upstream of *crp* gene. The transcription initiation resulted in primary transcripts under the specified conditions. While *crp* is regulated in the same manner with two major σ factors, *cutA* represented altered σ factor binding and changed transcription unit structure. In both species, *cutA* is located between *dcuA* and *dipZ* orthologous genes in the lagging strand. However, those 3 genes were expressed in a contiguous transcript, indicating one transcription unit. The first gene, *dcuA*, was bound by σ^70^ and had two TSSs. On the other hand, in *K. pneumoniae*, *cutA* was directly bound by σ^70^ and *dcuA* was not. Although no TSS was identified upstream of *cutA* in *K. pneumoniae*, a sharp edge at the transcription starting point indicated a transcription initiation from that particular gene. *panD* showed another noteworthy example of disruption in the transcription unit and change in σ factor association, in two closely related microorganisms. In *E. coli*, *yadD* got in between *panD* and *panC*, resulting in 3 transcription units, while *K. pneumoniae* had an intact gene order and one transcription unit. In *K. pneumoniae*, *panBCD* made one transcription unit, supported by expression profiling and TSS dataset, and was bound by σ^70^ under exponential phase. However, interestingly, there were 3 distinct transcription units, *panD*, *yadD,* and *panBC*, which were expressed and regulated respectively. Their separate expression was confirmed by expression profiling and TSS determination, and *panD* and *panBC* transcription units were both bound by σ^70^. *ydeA* represents a case where σ factor binding is swapped between σ^70^ and σ^38^. *ydeA* and *marC* are adjacent in both genomes. However, *ydeA* lies on the leading strand in *E. coli*, while it is on the lagging strand in *K. pneumoniae*. Interestingly, it is regulated by σ^38^ in *E. coli*; however it is bound by σ^70^ in *K. pneumoniae*.

**Statistics on TSSs**

TSSs for 4 conditions, exponential phase, stationary phase, alternative nitrogen source, and heat shock, were analyzed in this study. Only TSSs that lay within promoter regions identified with ChIP-chip datasets were kept, and this resulted in 4,724 TSSs associated with RNAP and/or σ-factors. Identified TSSs showed no strand preference, with 48.2% on the leading strand and 51.8% on the lagging strand. As reported previously[[3](#_ENREF_3)], purine (A and G) was preferred at TSS/+1 sites, whereas pyrimidine (T and C) was used more at -1 sites (**Figure S6**). Among 4,724 TSSs, 48.5% were identified in more than two conditions, indicating that a large fraction of TSSs and their promoters were active in transcription initiation under multiple conditions (**Figure S7**).

**SUPPLEMETAL REFERENCES**

1. Farewell A, Kvint K, Nystrom T: **Negative regulation by RpoS: a case of sigma factor competition**. *Mol Microbiol* 1998, **29**(4):1039-1051.

2. Loewen PC, Hu B, Strutinsky J, Sparling R: **Regulation in the rpoS regulon of Escherichia coli**. *Can J Microbiol* 1998, **44**(8):707-717.

3. Kim D, Hong JS, Qiu Y, Nagarajan H, Seo JH, Cho BK, Tsai SF, Palsson BO: **Comparative analysis of regulatory elements between Escherichia coli and Klebsiella pneumoniae by genome-wide transcription start site profiling**. *PLoS genetics* 2012, **8**(8):e1002867.

4. Datsenko KA, Wanner BL: **One-step inactivation of chromosomal genes in Escherichia coli K-12 using PCR products**. *Proceedings of the National Academy of Sciences of the United States of America* 2000, **97**(12):6640-6645.

5. Powell BS, Court DL, Inada T, Nakamura Y, Michotey V, Cui X, Reizer A, Saier MH, Jr., Reizer J: **Novel proteins of the phosphotransferase system encoded within the rpoN operon of Escherichia coli. Enzyme IIANtr affects growth on organic nitrogen and the conditional lethality of an erats mutant**. *The Journal of biological chemistry* 1995, **270**(9):4822-4839.

6. Cho BK, Zengler K, Qiu Y, Park YS, Knight EM, Barrett CL, Gao Y, Palsson BO: **The transcription unit architecture of the Escherichia coli genome**. *Nature biotechnology* 2009, **27**(11):1043-1049.

7. Herring CD, Raffaelle M, Allen TE, Kanin EI, Landick R, Ansari AZ, Palsson BO: **Immobilization of Escherichia coli RNA polymerase and location of binding sites by use of chromatin immunoprecipitation and microarrays**. *Journal of bacteriology* 2005, **187**(17):6166-6174.

8. Cho BK, Barrett CL, Knight EM, Park YS, Palsson BO: **Genome-scale reconstruction of the Lrp regulatory network in Escherichia coli**. *Proceedings of the National Academy of Sciences of the United States of America* 2008, **105**(49):19462-19467.

9. Cho BK, Knight EM, Barrett CL, Palsson BO: **Genome-wide analysis of Fis binding in Escherichia coli indicates a causative role for A-/AT-tracts**. *Genome research* 2008, **18**(6):900-910.

10. Salgado H, Peralta-Gil M, Gama-Castro S, Santos-Zavaleta A, Muniz-Rascado L, Garcia-Sotelo JS, Weiss V, Solano-Lira H, Martinez-Flores I, Medina-Rivera A *et al*: **RegulonDB v8.0: omics data sets, evolutionary conservation, regulatory phrases, cross-validated gold standards and more**. *Nucleic acids research* 2013, **41**(Database issue):D203-213.

11. Cho BK, Federowicz S, Park YS, Zengler K, Palsson BO: **Deciphering the transcriptional regulatory logic of amino acid metabolism**. *Nature chemical biology* 2012, **8**(1):65-71.
